# Global CO_2_ fertilization of *Sphagnum* peat mosses via suppression of photorespiration during the twentieth century

**DOI:** 10.1038/s41598-021-02953-1

**Published:** 2021-12-31

**Authors:** Henrik Serk, Mats B. Nilsson, Elisabet Bohlin, Ina Ehlers, Thomas Wieloch, Carolina Olid, Samantha Grover, Karsten Kalbitz, Juul Limpens, Tim Moore, Wiebke Münchberger, Julie Talbot, Xianwei Wang, Klaus-Holger Knorr, Verónica Pancotto, Jürgen Schleucher

**Affiliations:** 1 Department of Medical Biochemistry and Biophysics, Umeå University, Umeå, Sweden; 2 Department of Forest Ecology and Management, Swedish University of Agricultural Sciences, Umeå, Sweden; 3 Department of Ecology and Environmental Sciences, Umeå University, Umeå, Sweden; 4 Department of Applied Chemistry and Environmental Science, RMIT University, Melbourne, Australia; 5 Institute of Soil Science and Site Ecology, Dresden University of Technology, Tharandt, Germany; 6 Department of Environmental Sciences, Wageningen University, Wageningen, The Netherlands; 7 Department of Geography, McGill University, Montreal, Canada; 8 Institute of Landscape Ecology, University of Münster, Munster, Germany; 9 Department of Geography, Université de Montréal, Montreal, Canada; 10 Northeast Institute of Geography and Agroecology, Chinese Academy of Sciences, Changchun, People’s Republic of China; 11 Centro Austral de Investigaciones Científicas (CADIC-CONICET), Ushuaia, Argentina

**Keywords:** Biochemistry, Biophysics, Chemical biology, Ecology, Plant sciences, Biogeochemistry, Climate sciences, Ecology, Environmental sciences

## Abstract

Natural peatlands contribute significantly to global carbon sequestration and storage of biomass, most of which derives from *Sphagnum* peat mosses. Atmospheric CO_2_ levels have increased dramatically during the twentieth century, from 280 to > 400 ppm, which has affected plant carbon dynamics. Net carbon assimilation is strongly reduced by photorespiration, a process that depends on the CO_2_ to O_2_ ratio. Here we investigate the response of the photorespiration to photosynthesis ratio in *Sphagnum* mosses to recent CO_2_ increases by comparing deuterium isotopomers of historical and contemporary *Sphagnum* tissues collected from 36 peat cores from five continents. Rising CO_2_ levels generally suppressed photorespiration relative to photosynthesis but the magnitude of suppression depended on the current water table depth. By estimating the changes in water table depth, temperature, and precipitation during the twentieth century, we excluded potential effects of these climate parameters on the observed isotopomer responses. Further, we showed that the photorespiration to photosynthesis ratio varied between *Sphagnum* subgenera, indicating differences in their photosynthetic capacity. The global suppression of photorespiration in *Sphagnum* suggests an increased net primary production potential in response to the ongoing rise in atmospheric CO_2_, in particular for mire structures with intermediate water table depths.

## Introduction

Over one third of global soil carbon (C) is stored in boreal mires^1,2^, making peat C accumulation an essential part of the global C budget. Changes in climate are expected to have strong effects on peatland C sequestration^1,3,4^. During the early and mid-Holocene, the accumulation of peat C was largely determined by the retreat of the northern ice sheet and the rise in temperature because atmospheric CO_2_ concentrations were relatively stable at 275 ± 8 ppm (SD)^5–7^. Since the beginning of the industrial revolution in the early nineteenth century, CO_2_ concentrations have risen from ca. 280 ppm to over 400 ppm today^8^. Multiple observations indicate that recent increases in atmospheric CO_2_ have affected peat C accumulation rates: (i) the variation in acrotelm peat accumulation was mainly driven by photosynthesis^9^, (ii) peat C accumulation in Alaskan mires increased about threefold during the twentieth century^10^, and (iii) the variation in net ecosystem exchange between mires was mainly controlled by differences in leaf area index^11^. In addition to rising atmospheric CO_2_ levels, ongoing climatic changes such as increases in temperature and changes in precipitation are hypothesized to influence peatland C fluxes^12–14^.

*Sphagnum* peat mosses are primarily responsible for the accumulation of peat C because they often constitute 80–100% of the ground cover in northern peatlands^15^. Compared to vascular plants, *Sphagnum* remnants are highly resistant to microbial decay, which is vital for peat C accumulation^16,17^. Therefore, C accumulation and storage in the form of *Sphagnum* remains generally exceeds C losses from microbial decay. However, currently it is not clear whether increases in *Sphagnum* C accumulation driven by ongoing and projected global warming will outweigh increases in the rate of microbial peat decomposition^9,18,19^. Understanding how *Sphagnum* C fluxes respond to recent and projected increases in atmospheric CO_2_ is therefore crucial for predicting future peat C fluxes.

To our knowledge, responses of *Sphagnum* photosynthetic C fluxes to the recent increase in atmospheric CO_2_ have never been explored on the global scale. Previous attempts to estimate responses of *Sphagnum* to increased atmospheric CO_2_ were either based on free-air CO_2_ enrichment (FACE) or greenhouse experiments^20–25^. A recently developed isotopomer method^26,27^ enables reconstruction of metabolic C fluxes by analyzing cell wall carbohydrates from *Sphagnum* remnants. This approach involves using NMR spectroscopy to measure the abundance ratio of the deuterium (D) isotopomers D6^*S*^ and D6^*R*^ in the C6H_2_ groups of glucose derived from hydrolyzed cell wall carbohydrates, where *S* and *R* are the stereochemical designators. The abundance ratio of these D isotopomers is correlated with the ratio of Rubisco oxygenation to carboxylation due to D fractionation in the photorespiration pathway^26^. Therefore, the D6^*S*^/D6^*R*^ ratio reflects the relative rates of photorespiration and (gross) photosynthesis which essentially depend on the substrate ratio of CO_2_ and O_2_. The uptake of C in C_3_ plants is facilitated by the carboxylation reaction, catalyzed by the enzyme Rubisco. The oxygenation activity of this enzyme, however, leads to the loss of C and therefore reduces the efficiency of photosynthesis. This process is known as photorespiration, which accounts for C losses of up to 25%, and therefore plays an important role for the global terrestrial C sink^28^.

Most studies focus on photorespiration in higher plants and only little is known about *Sphagnum* mosses, despite their importance for global peatland C fluxes. Using the isotopomer method, we recently experimentally investigated the response of the photorespiration to photosynthesis ratio (*i.e.* the D6^*S*^/D6^*R*^ ratio) to the recent increase in atmospheric CO_2_ levels from 280 to 400 ppm and the dependence of this response on selected climate variables including temperature, water table (WT) depth, and light intensity^27^. We found that under low WT conditions (20 cm below moss surface), photorespiration was suppressed relative to photosynthesis in the hummock species *Sphagnum fuscum*. Under water-saturating conditions (WT near moss surface), however, there was no effect of atmospheric CO_2_, indicating that WT depth strongly influences the CO_2_ fertilization effect in *S. fuscum*. In addition, the lawn species *S. majus* did not respond to the CO_2_ increase, suggesting a species-specific response. Therefore, *Sphagnum* photosynthetic C fluxes are expected to vary with species and microhabitat^29–32^.

**Figure 1:**
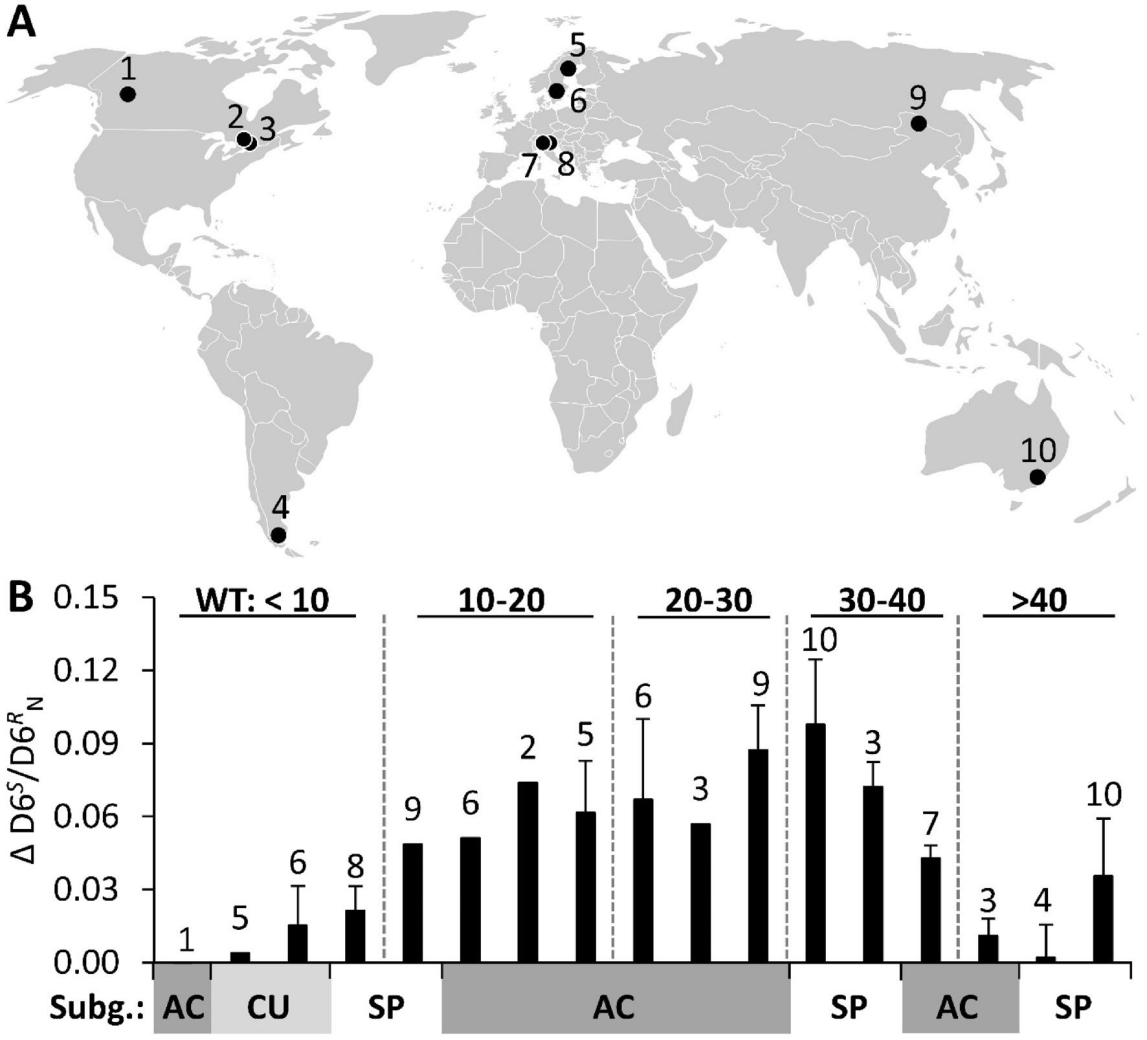
Global changes in the deuterium isotopomer ratio (ΔD6^*S*^*/*D6^*R*^_N_) of *Sphagnum* during the twentieth century representing changes in the photorespiration to photosynthesis ratio. (**A**) Global distribution of investigated sites. (**B**) Response of the D6^*S*^/D6^*R*^ ratio per unit change in 1000/[CO_2_] between modern and historical *Sphagnum* samples (ΔD6^*S*^*/*D6^*R*^_N_). Five water table depths (WT) categories (in cm) are indicated by vertical dashed lines. *Sphagnum* subgenera are indicated on the x-axis by grey/white shading: AC, ACUTIFOLIA (dark grey); CU, CUSPIDATA (light grey); SP, SPHAGNUM (white). Error bars indicate standard error, n = 1–4 (see Table S1 for more information). Numbers above error bars correspond to sample sites as numbered in (**A**).

The aim of this study is to investigate changes in *Sphagnum* photosynthetic C fluxes during the twentieth century at the global scale. To do this, we estimated the global response of the photorespiration to photosynthesis ratio by comparing the D6^*S*^/D6^*R*^ ratios of modern *Sphagnum* tissues formed at the current atmospheric CO_2_ concentration (ca. 400 ppm) to the D6^*S*^/D6^*R*^ ratios of *Sphagnum* remnants from peat core sections formed at least 100 years ago under pre-industrial CO_2_ concentrations (≤ 300 ppm). Our analysis is based on 36 peat cores from 10 different sites on five continents (Fig. 1A). Effects of microhabitat were tested by including both hummock (n = 25) and lawn (n = 11) samples with WT depths ranging from ≈ 5 to 70 cm below the moss surface. The effect of species was tested by including eight different *Sphagnum* species belonging to three different taxonomic sections (subgenera). To determine whether the CO_2_ response of the photorespiration to photosynthesis (D6^*S*^/D6^*R*^) ratio in *Sphagnum* was influenced by changes in temperature and precipitation during the twentieth century, we estimated these changes using established climate models^33–35^. The effects of changes in WT depth during the twentieth century were estimated using WT reconstruction data available in the literature and by measuring δ^13^C, which has been proposed as proxy for surface moisture^27,36^.

## Results

### Global suppression of photorespiration during the twentieth century

Global changes in the photorespiration to photosynthesis ratio during the twentieth century were assessed by comparing the D6^*S*^*/*D6^*R*^ ratios of modern and ≥ 100 years old *Sphagnum* tissues. Modern *Sphagnum* samples were retrieved from surface peat (top 0–2 cm) formed at contemporary atmospheric CO_2_ levels (ca. 400 ppm). Conversely, historical *Sphagnum* tissues were retrieved from peat layers ≥ 30 cm below the surface, with an approximate age of 100 years or more (Table S1). Therefore, the historical *Sphagnum* tissues were formed when atmospheric CO_2_ concentrations were ≤ 300 ppm. The D6^*S*^*/*D6^*R*^ ratio of modern *Sphagnum* was 0.860 ± 0.004 (average ± SE, range: 0.810–0.927), while that of historical *Sphagnum* was 0.901 ± 0.005 (average ± SE, range: 0.858–0.971, Fig. S1). Thus, the D6^*S*^*/*D6^*R*^ ratio of modern *Sphagnum* was generally lower compared to ≥ 100 years old *Sphagnum*, indicating that photorespiration is suppressed relative to photosynthesis.

The peat samples essentially differed with respect to their depth below the surface (which is related to the atmospheric CO_2_ concentration when the *Sphagnum* biomass was formed), the *Sphagnum* subgenus, the present WT depth, and the geographical location (site). The effects of these factors on the D6^*S*^/D6^*R*^ ratio were tested by a linear mixed effect model with the site as random factor, and atmospheric CO_2_, WT and subgenus as fixed factors. Analysis of variance (ANOVA) of the random factor revealed that the effect of site was not significant (variance = 0.0, *p* = 1.0). ANOVA of the fixed factors showed a significant effect for atmospheric CO_2_, WT and subgenus, explaining 35%, 12% and 8% of the variance, respectively (Table 1). A significant interaction was found between CO_2_ and WT, which explained 12% of variance, indicating that the effect of CO_2_ on the D6^*S*^/D6^*R*^ ratio is dependent on the WT depth.

**Table 1:** Summary of ANOVA results for linear mixed effect models of the effects of atmospheric CO_2_, water table (WT) and *Sphagnum* subgenus (Subg.) on the D6^*S*^/D6^*R*^ ratio.

Factor	*Df*	F	*p*	*R*^2^
CO_2_	1, 62	59.75	< 0.001	0.35
WT	4, 62	4.87	0.002	0.12
Subg	2, 62	6.36	0.004	0.08
CO_2_ × WT	4, 62	5.05	0.002	0.12
CO_2_ × Subg	*n.s.*			
WT × Subg	*n.s.*			
CO_2_ × WT × Subg	*n.s.*			

*Df*, degrees of freedom of the model and residuals. Non-significant factors/interactions with *p* > 0.1 are denoted as *n.s.*

The effect of atmospheric CO_2_ on the D6^*S*^/D6^*R*^ ratio clearly depended on the present WT depth. The post-hoc test showed that for peat cores where the WT was < 10 cm below the moss surface, the D6^*S*^/D6^*R*^ ratios were not significantly different (*p* > 0.05, Fig. S1) between modern and historical *Sphagnum*, with means of 0.893 ± 0.01 and 0.905 ± 0.009 (SE), respectively. Conversely, for peat cores with WT depths of 10–40 cm, the D6^*S*^/D6^*R*^ ratios of modern and historical *Sphagnum* clearly differed (*p* < 0.05), having means of 0.850 ± 0.004 and 0.910 ± 0.007 (SE), respectively (Figs. 1 and S1). For peat cores with WT depths > 40 cm, the difference between modern and historical samples was again not significant (*p* > 0.05), with means of 0.860 ± 0.007 and 0.873 ± 0.004 (average ± SE), respectively.

For modern *Sphagnum*, the effect of WT was evident from generally higher D6^*S*^/D6^*R*^ ratios for WT depths of < 10 cm (mean 0.893 ± 0.01 SE), compared to samples with WT depths of > 10 cm (mean 0.852 ± 0.007 SE; Fig. S1). For ≥ 100 years-old samples, this was not the case, instead the D6^*S*^/D6^*R*^ ratio was generally lower for WT depths > 40 cm (mean 0.873 ± 0.004 SE) compared to WT depths of < 40 cm (mean 0.910 ± 0.01 SE). The *Sphagnum* subgenus also had a clear effect: the mean D6^*S*^/D6^*R*^ ratios for modern and historical samples of ACUTIFOLIA species were 0.858 ± 0.004 and 0.906 ± 0.007 (SE), respectively, while those for species of the subgenus SPHAGNUM were 0.846 ± 0.006 and 0.885 ± 0.006 (SE), respectively. The D6^*S*^/D6^*R*^ ratio was thus generally lower for species of the subgenus SPHAGNUM than for ACUTIFOLIA species. Modern and historical samples of CUSPIDATA species had higher mean D6^*S*^/D6^*R*^ ratios of 0.911 ± 0.008 and 0.921 ± 0.009 (SE), respectively, with no significant difference (*p* > 0.05) between modern and historical samples (Figs. 1 and S1).

The differences in the D6^*S*^/D6^*R*^ ratio between modern and historical *Sphagnum* were normalized based on the linear relationship between the D6^*S*^*/*D6^*R*^ ratio and 1000/[CO_2_] previously reported by Ehlers et al.^26^, to account for variations in atmospheric CO_2_ concentrations due to differences in peat depth and/or age. To this end, the regression slope of this linear function was calculated as the change in the D6^*S*^*/*D6^*R*^ ratio per unit change in 1000/[CO_2_] between modern and historical *Sphagnum* samples (denoted ΔD6^*S*^*/*D6^*R*^_N_). ΔD6^*S*^*/*D6^*R*^_N_ thus represents the degree of suppression of photorespiration; its mean was 0.044 ± 0.008 (SE) and it varied between 0.000 and 0.094 (Fig. 1). ΔD6^*S*^*/*D6^*R*^_N_ varied with the WT depth: it was 0.010 ± 0.005 (average ± SE) for WT depths < 10 cm below the moss surface, 0.066 ± 0.006 (average ± SE) for WT depths between 10 and 40 cm, and 0.016 ± 0.010 (average ± SE) for WT depths > 40 cm (Fig. 1). These results indicate that a WT between 10 and 40 cm below the moss surface is optimal for suppressing photorespiration in response to increased atmospheric CO_2_.

### Effect of changes in water table, temperature and precipitation during the twentieth century

The relationship between ΔD6^*S*^*/*D6^*R*^_N_ and the present WT depth assumes that hydrological conditions were relatively stable over the twentieth century. To test this assumption, we performed a literature review on changes in WT depth during the twentieth century based on previously published testate amoebae WT reconstruction data (Table 2). These literature data were mostly from hummock peat cores with WT depth > 10 cm (except site 1, WT = 8 cm). In cases where data were not available for the sampled mires, data were retrieved for mires from the same region (Tables 2 and S2), assuming that the similarities in regional climate would result in similar changes in WT depth^37^. Additionally, variations in other climate parameters such as temperature and precipitation may have affected the suppression of photorespiration (ΔD6^*S*^*/*D6^*R*^_N_). Therefore, we also estimated changes in temperature and precipitation during the twentieth century using established climate models^33–35^ (Table 2).

**Table 2:** Historical changes in water table depth (ΔWT), mean annual air temperature (ΔMAT) and total annual precipitation (ΔTAP) during the twentieth century at the sites shown in Fig. 1.

Site	ΔWT (cm)	ΔMAT (°C)	ΔTAP (%)	Reference (WT)
1	≈ 0	+ 3.1	+ 9.9	^38^
2, 3	+ 4	+ 0.2	− 6.7	^39,40^
4	− 40*	− 1.7	− 32.6	^41^
5	≈ 0*	+ 2.8	+ 19.1	^37^
6	− 5*	+ 1.3	+ 11.1	^37,42^
7, 8	− 11*	+ 0.4	+ 12.5	^37^
9	− 6	+ 1.6	+ 25.0	^43^
10	− 30*	+ 0.1	+ 15.1	^44^

Changes in TAP are specified in percent change. (−) indicates a decrease and (+) indicates an increase. (*) indicates WT data for another mire in the same region as the relevant site (Table S2).

**Figure 2:**
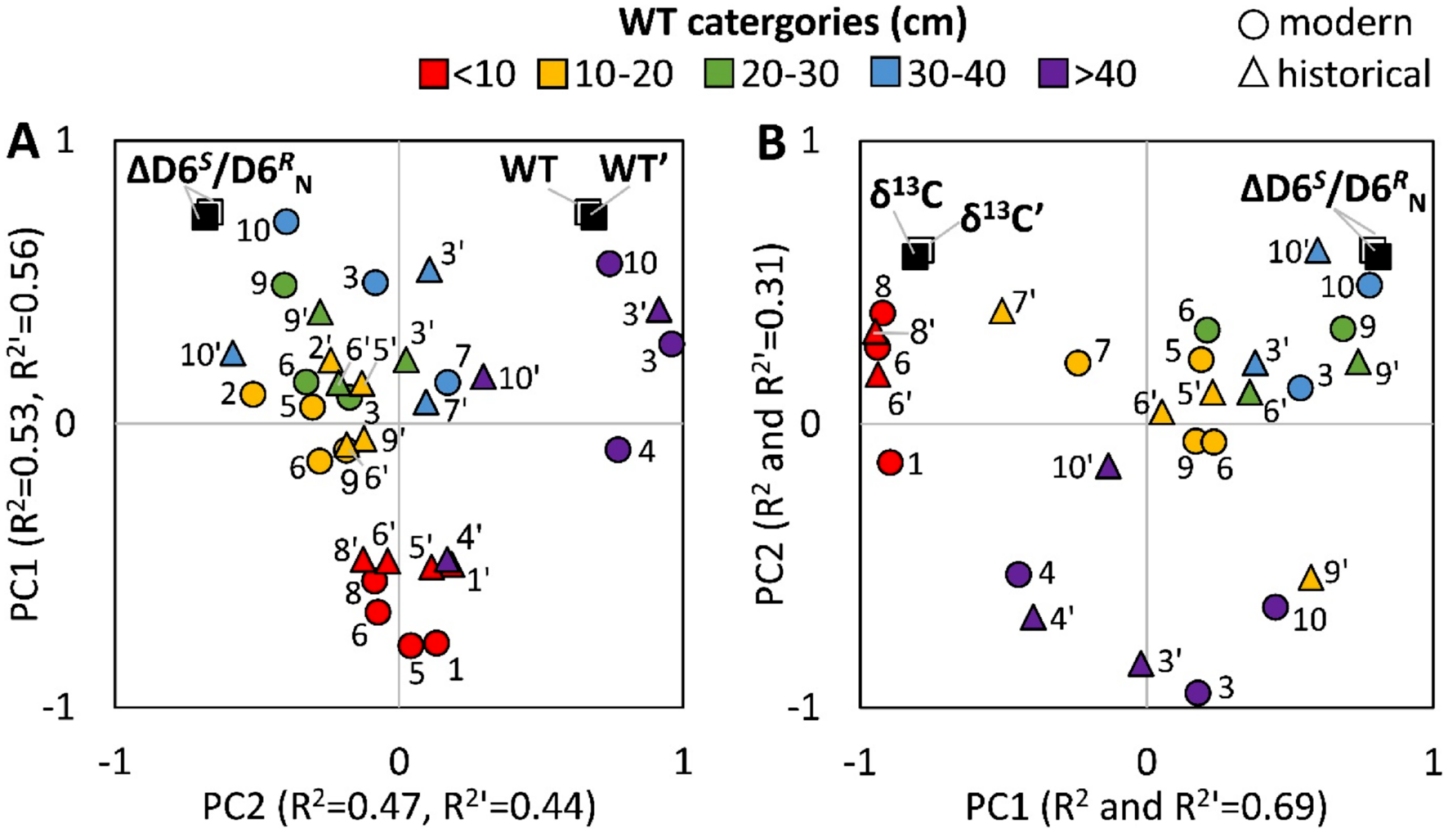
PCA biplots of the deuterium isotopomer ratio (ΔD6^*S*^/D6^*R*^_N_) of *Sphagnum* during the twentieth century and (**A**) the modern water table depth (WT, circles) and the historical water table depth inferred from testate amoebae data from literature (WT′, triangles), and (**B**) modern *Sphagnum*-δ^13^C (δ^13^C) and historical δ^13^C data of ≥ 100 years-old *Sphagnum* samples (δ^13^C′). Colour coding indicates WT categories. Numbers indicate respective sites in Fig. 1A. Apostrophe indicates historical data point. Note that in (**B**) δ^13^C data for site 2, site 3 (WT 20–30 cm) and site 5 (WT > 10 cm) are missing due to insufficient sample material.

Changes in WT during the twentieth century were generally small (between + 4 and − 11 cm), except in southern Argentina and Australia, where the WT depth decreased by 40 and 30 cm, respectively (Table 2). To determine whether changes in historical climate data contribute to the ΔD6^*S*^*/*D6^*R*^_N_-response, we performed a three-way ANOVA, with the change in WT (ΔWT), mean annual air temperature (ΔMAT), and total annual precipitation (ΔTAP) as factors. No significant effect of ΔWT, ΔMAT and ΔTAP could be detected (*p* > 0.2). However, a significant interaction was found between ΔWT and ΔMAT (*R*^2^ = 0.16, F = 53.8, *p* = 0.028). Further, we tested if specifically the changes in mean summer air temperature (ΔMSAT) or the total summer precipitation (ΔTSP) affect the ΔD6^*S*^*/*D6^*R*^_N_–response by performing a three-way ANOVA with ΔWT, ΔMSAT and ΔTSP as factors. Again, no significant effect could be detected (*p* > 0.2), except for an interaction between ΔWT and ΔMSAT (*R*^2^ = 0.17, F = 53.3, *p* = 0.029). Thus, a small part of the variation in ΔD6^*S*^*/*D6^*R*^_N_ (17%) of *Sphagnum* may be explained by combined changes in WT and temperature during the twentieth century. Additionally, we performed principal component analysis (PCA) to compare the relationship between the ΔD6^*S*^*/*D6^*R*^_N_–response and the modern WT depth, with historical WT depths from literature data (Fig. 2A). The results show that the general pattern remains the same for both modern and historical WT depths, indicating that the observed ΔD6^*S*^*/*D6^*R*^_N_–response is not due to changes in WT during the twentieth century.

### *Sphagnum* δ^13^C as proxy for changes in water table depth

The carbon isotopic signature (δ^13^C) of *Sphagnum* peat has been proposed as proxy for surface moisture^27,36,45,46^. Therefore, we tested the use of δ^13^C as a potential indicator of changes in WT depth by measuring the δ^13^C of both modern and ≥ 100 years-old whole-*Sphagnum* tissues. Regression analysis revealed a highly significant correlation between δ^13^C in modern *Sphagnum* and the present WT depth (*R*^2^ = 0.67, *p* < 0.001, Fig. S3A) confirming that δ^13^C reflects changes in WT depth. The δ^13^C values became more negative (*i.e.* more depleted) with increasing WT depth, from − 25.8 ± 0.2‰ (average ± SE) at WT depths < 10 cm to − 30.3 ± 0.5‰ (average ± SE) at WT depths > 60 cm below surface. For ≥ 100 years-old *Sphagnum*, δ^13^C also correlated significantly with the historical WT depth inferred from testate amoebae data in Table 2 (*R*^2^ = 0.31, *p* < 0.002; Fig. S3B). Thus, both modern and historical *Sphagnum* showed the same trend (with slopes of 0.07 and 0.05, respectively), indicating that the WT depth ≥ 100 years ago was similar to that today. The PCA further confirmed that the pattern between the ΔD6^*S*^/D6^*R*^_N_-response and modern *Sphagnum*-δ^13^C values remained the same for the historical δ^13^C values from ≥ 100 years-old *Sphagnum* samples (Fig. 2B). These results support that changes in WT during the twentieth century were generally small for the sites investigated, and not relevant to the isotopomer response.

## Discussion

Our results show that the increase in atmospheric CO_2_ during the twentieth century suppressed photorespiration relative to C assimilation in *Sphagnum* mosses, thus increasing the potential net photosynthesis. However, this suppression is strongly dependent on the moisture status of the moss. High moisture contents, typical for *Sphagnum* grown at WT depths < 10 cm, resulted in no significant suppression of photorespiration, with a mean ΔD6^*S*^*/*D6^*R*^_N_ value of 0.010 (*p* > 0.05; Figs. 1, and S1). In contrast, the mean ΔD6^*S*^*/*D6^*R*^_N_ for WT depths between 10 and 40 cm was 0.066 (*p* < 0.05), clearly reflecting a strong suppression of photorespiration. This latter response is identical to that obtained from CO_2_ manipulation experiments with higher C_3_ plants and corresponds to an increase in net photosynthesis of 35%, assuming constant ribulose 1,5-bisphosphate (RuBP) turnover rates^26,47^. The similarity of the kinetic properties of Rubisco between different C_3_ plants (including mosses)^48^, suggests that the observed response in *Sphagnum* also corresponds to an increase in net photosynthesis of 35%. Conversely, the low ΔD6^*S*^*/*D6^*R*^_N_ value for *Sphagnum* grown at WT < 10 cm indicates no significant increase in net photosynthesis. This suggests that hollow and lawn *Sphagnum* communities that experience high WT do not profit from CO_2_ fertilization. However, CO_2_ fertilization is beneficial in mire structures that experience intermediate WT depths, mostly hummocks, and may therefore stimulate hummock formation and topographic development as atmospheric CO_2_ concentrations rise.

Some factors may limit the CO_2_ fertilization effect in hummocks. In particular, our data show that a very low WT (> 40 cm) has a negative effect on CO_2_ fertilization, *i.e.* on the suppression of photorespiration: the mean ΔD6^*S*^*/*D6^*R*^_N_ value for samples in the > 40 cm WT group was 0.016 (*p* > 0.05; Figs. 1, and S1). This indicates that *Sphagnum* mosses do not respond to increased atmospheric CO_2_ under water limiting conditions, *i.e.* drought. Climate modelling of peatland C fluxes indicates that *Sphagnum* gross primary production decreases significantly at WT > 40 cm^49^. Concomitantly, increased releases of CO_2_ from sub-surface peat decomposition during drought^50,51^ may decouple the mosses’ response to changes in atmospheric CO_2_.

The D6^*S*^*/*D6^*R*^ ratio of *S. fuscum* responded strongly to experimental manipulation of the CO_2_ concentration^27^, rising by 0.03 on average (ΔD6^*S*^*/*D6^*R*^_N_) when CO_2_ levels increased by 120 ppm at a WT of 20 cm. In *S. fuscum* samples from peat cores with WT depths > 10 cm, the suppression of photorespiration (ΔD6^*S*^*/*D6^*R*^_N_) was 0.06 on average in response to a CO_2_ concentration increase of ca. 100 ppm (Fig. 1). This suggests that the response to changes in atmospheric CO_2_ levels under field conditions has been stronger than in growth chambers. In the field, environmental conditions such as light and nutrient levels may differ from the growth chamber conditions^52,53^. Whether these factors affect the CO_2_-driven suppression of photorespiration requires further investigation.

The peat core data in this study showed that higher CO_2_ concentrations did not cause any suppression of photorespiration in *S. majus* grown at typical WT depths close to the mire surface (ΔD6^*S*^*/*D6^*R*^_N_: 0.00, Fig. 1). This result is consistent with the response observed in CO_2_ manipulation experiments with *S. majus* for WT levels of 0 and 7 cm (ΔD6^*S*^*/*D6^*R*^_N_: 0.00)^27^. Overall, the growth chamber experiments showed that water-saturating conditions prevent the suppression of photorespiration in both hummock and lawn *Sphagnum* species. The responses of the D6^*S*^*/*D6^*R*^ ratio and δ^13^C in our global dataset (Figs. 1, 2, S1, S2) are consistent with these results and demonstrate that a WT depth deeper than 10 cm is needed to maximize photosynthetic C fluxes in *Sphagnum.* Under these conditions, the mosses have the strongest potential to respond to the CO_2_ fertilization effect.

The ANOVA (Table 1) showed a significant effect of the subgenus on the D6^*S*^/D6^*R*^ ratio. Species of the section SPHAGNUM had generally lower D6^*S*^/D6^*R*^ ratios than ACUTIFOLIA species, suggesting that the relative rate of photorespiration is lower in SPHAGNUM species. The ratio of photorespiration to photosynthesis is directly related to the intracellular CO_2_ concentration (*c*_i_) at the site of Rubisco carboxylation^54^, suggesting differences in *c*_i_ between these two subgenera. Distinct leaf anatomical traits are responsible for different water holding capacities of these two subgenera^55^, and potentially influence *c*_i_. Altogether, this indicates that species of the subgenus SPHAGNUM have higher photosynthetic capacities than ACUTIFOLIA species, assuming that the RuBP turnover rates are similar.

Concerning the CO_2_-driven suppression of photorespiration, both ACUTIFOLIA and SPHAGNUM species showed a high suppression of photorespiration, and no difference in this response between these two subgenera (average ΔD6^*S*^/D6^*R*^_N_ = 0.05 for both). Thus, no significant interaction was found between CO_2_ and subgenus (Table 1). In contrast, species of the subgenus CUSPIDATA showed no suppression of photorespiration (Fig. 1). Unlike the ACUTIFOLIA and SPHAGNUM samples (WT range 8 to 67 cm, both), the CUSPIDATA samples came from high WT cores (WT ca. 5 cm), making it impossible to determine whether this response is species-specific. However, CUSPIDATA species require high WT levels to maintain growth^56^, suggesting that they will not respond to changes in atmospheric CO_2_ in any case.

Our dataset revealed that the extent of the CO_2_-driven suppression of photorespiration in *Sphagnum* mosses depends on the WT depth at the time of moss photosynthesis. To exclude potential effects of changes in WT during the twentieth century on the suppression of photorespiration, we estimated historical changes in WT by both measured *Sphagnum*-δ^13^C data and published testate amoebae data (Table 2; Fig. 2). Both datasets revealed no relationship between the observed suppression of photorespiration and historical changes in WT. These results support our assumption that changes in hydrological conditions during the twentieth century did not attenuate the suppression of photorespiration caused by rising atmospheric CO_2_ levels.

Moreover, we tested potential effects of changes in temperature and precipitation during the twentieth century on the CO_2_-driven suppression of photorespiration (ΔD6^*S*^/D6^*R*^_N_). Our data did not indicate any relationship between the changes in temperature or precipitation during the twentieth century and the suppression of photorespiration (Table 2), supporting that these changes do not attenuate the observed response. The absence of an effect of temperature on the suppression of photorespiration is consistent with results from climate chamber experiments^27^, where a temperature increase of 5 °C did not affect the magnitude of suppressed photorespiration. However, the photorespiration to photosynthesis (D6^*S*^/D6^*R*^) ratio increased slightly with elevated temperature (0.002 °C^−1^)^27^. Thus, the concomitant increase in temperature with atmospheric CO_2_ during the twentieth century may have reduced the CO_2_-driven suppression of photorespiration by approximately 0.002 units (for a temperature increase of 1 °C)^8^.

On the molecular scale, the D6^*S*^/D6^*R*^ ratio reflects changes in the Rubisco oxygenation to carboxylation flux ratio. Here we extended this molecular model to study global responses in these metabolic C fluxes to environmental drivers in *Sphagnum*. Our results indicate that ongoing increases in atmospheric CO_2_ suppress photorespiration relative to C assimilation in *Sphagnum.* According to photosynthesis models, this suppression may increase C uptake by up to 35% (depending on the WT level), which points towards an increase in net primary production (NPP) during the twentieth century. However, NPP is influenced by many other factors such as temperature, precipitation and sink limitations^9,57^. Modelling of peatland C dynamics^49,58^ suggests that temperature and precipitation have opposing influences on the C uptake response to the recent increase in atmospheric CO_2_. An increase in temperature combined with a decrease in precipitation reduces C uptake, whereas a small increase in temperature combined with a large increase in precipitation results in enhanced C uptake. We found a significant interaction between the mean annual/summer temperature and WT during the twentieth century, indicating a possible link between the observed changes in metabolic fluxes on the molecular level and global peat C assimilation. Thus, upscaling of the isotopomer data to global responses in peat C fluxes provide valuable information for the mechanistic understanding of photosynthetic responses of *Sphagnum* mosses to ongoing and future climate changes.

## Conclusion

Here we used deuterium isotopomers to study global responses in photosynthetic C fluxes in *Sphagnum* mosses to the twentieth century’s increase in atmospheric CO_2_. This method allowed us to upscale changes in metabolic C flux ratios from the molecular level to the global scale. We were able to track historical changes of metabolic C fluxes over long time scales using peat archives and link these results to observations from short-term manipulation experiments. Thus, our results will help to: (i) develop mechanistic models of global metabolic C fluxes, (ii) assess the role of peatlands for the global C budget during the twentieth century, and (iii) improve the prediction of future responses of peatlands to increases in atmospheric CO_2_ and climate change. Furthermore, our results point out that mire structures with intermediate WT depths, such as hummocks, will benefit strongly from CO_2_ fertilization, unlike lawn and hollow *Sphagnum* communities that often experience high WT depths.

## Materials and methods

### Plant material-peat cores

Hummock and lawn peat cores were retrieved from 10 sites located in Sweden (2), Italy (2), Canada (3), China (1), Argentina (1), and Australia (1) between 2014 and 2018. The sites’ latitudes ranged from 55°S to 64°N, their mean annual air temperatures from − 3.9 to 6.3 °C, mean annual precipitation levels from 369 to 1270 mm, and elevations from 35 to 1700 m asl. Detailed descriptions of each site are provided in the supplement (Table S3). Historical *Sphagnum* samples were retrieved from peat depths between 30 and 40 cm, except in the cores from southern Argentina (60 cm). All historical samples were found to be approximately 100 years or older, based on ^210^Pb radiometric dating or estimates obtained using published age-depth profiles for each site (Table S1). The atmospheric CO_2_ concentration when the historical *Sphagnum* samples formed was estimated for the respective year and ranged between 280 and 310 ppm (Table S1)^59^. Since no major changes in atmospheric CO_2_ levels occurred between year 0 and 1900, uncertainties in the age-estimates are not expected to have major effects on the estimated CO_2_ concentration.

Peat cores were extracted using a sharp knife or a peat corer, yielding cores with dimensions of 8 × 8 cm or diameters of 10 cm, respectively. Peat cores were either wrapped in plastic film and stored at − 20 °C or sliced into 2 cm sections, oven dried (at 60 °C), and transferred to Ziploc-bags before further transport to Sweden. In the case of frozen cores, the topmost and bottom-most 2 cm were sliced off and thawed, and vascular plant material and mosses other than *Sphagnum* were removed. In cases where no intact species could be retrieved from the bottom of the core (six cores), the material was washed through a series of sieves (mesh sizes: 3 mm, 1.6 mm, 0.8 mm and 0.5 mm). The residue of the 0.5 mm sieve consisted almost exclusively of *Sphagnum* leaves. *Sphagnum* species from the top and bottom of the core were identified using a stereomicroscope and a binocular microscope (100 and 400×). The species were identified according to literature^60–63^. Peat cores in which the dominant *Sphagnum* species/subgenus differed between top and bottom were excluded because such differences indicate a change in microtopography. After processing as described above, samples were dried at 60 °C for 3 days. The WT depth in Table S1 was measured at the time of sampling, which happened generally during the summer months. In order to consider local WT fluctuations, we compared the WT measurements with mean seasonal and testate amoebae inferred WT data from the literature for each site respectively (Table S4). A PCA biplot confirmed that the WT measurements match well with mean growing season WT depths reported in the literature (Fig. S4).

### Sample preparation for deuterium isotopomer measurements

The dried *Sphagnum* samples were ground to a fine powder at 30 Hz for 2 min using a MM 400 ball mill (Retsch^®^, Haan, Germany), and 200–700 mg portions were used to prepare samples for Deuterium isotopomer measurements. Glucose-containing structural polymers were hydrolyzed to glucose and converted to 1,2-O-isopropylidene-α-d-glucofuranose according to established protocols^64^. To remove contamination by a mannose derivative whose NMR signals overlap with those of the glucose derivative, an oxidation step was applied as previously described^27^. The derivative was subsequently converted into 3,6-anhydro-1,2-O-isopropylidene-α-d-glucofuranose following published procedures^65^. The latter compound was purified by flash chromatography using silica gel and diethyl ether. Pure fractions were identified by thin-layer-chromatography and pooled. Diethyl ether was evaporated, the sample was washed with amylene-stabilized chloroform, and its purity was checked by ^1^H-NMR.

### Deuterium isotopomer quantification

For NMR measurements of intramolecular deuterium abundances, each sample of the glucose derivative prepared as described above was dissolved in a mixture of 83% v/v acetonitrile, 17% C_6_F_6_, and 0.01% C_6_D_6_, then transferred to a 5-mm NMR tube with a PTFE valve (J. Young Scientific Glassware Ltd., Windsor, U.K.) containing ca. 5 mg of NaHCO_3_. Deuterium NMR spectra were acquired and processed as previously described^64^, using an AVANCE III 850 spectrometer (Bruker BioSpin GmbH, Rheinstetten, Germany) equipped with a ^19^F lock and a cryogenic probe optimized for deuterium detection. Deuterium NMR spectra were integrated by deconvolution with a Lorentzian line shape fit, using TopSpin™ 3.2 (Bruker BioSpin GmbH, Rheinstetten, Germany). The D6^*S*^/D6^*R*^ isotopomer ratio was determined as the ratio of the integrals of the D6^*S*^ and D6^*R*^ signals^26^. For each sample, five to eight spectra were recorded and the average D6^*S*^/D6^*R*^ ratio was calculated.

### C-isotope analysis

C-isotopic signatures (δ^13^C) of dry moss tissue samples (ca. 5 mg) were analyzed by conversion into CO_2_ by combustion and quantification by mass spectrometry^66^, using an elemental analyser (Flash EA 2000, Thermo Fisher Scientific, Bremen, Germany) coupled to an isotope ratio mass spectrometer (DeltaV, Thermo Fisher Scientific, Bremen, Germany). The data were corrected for drift and non-linear sample size effects. For quantification, we used laboratory standards consisting of wheat and maize flours calibrated against two certified δ^13^C reference standards: IAEA-CH-6, and USGS40.

### Statistical analysis

Prior to statistical analysis, the samples from the top and bottom of each core were assigned as high and low CO_2_ respectively. Based on their present WT depths, samples were divided into five different groups: < 10, 10–20, 21–30, 31–40 and > 40 cm below surface. Individual *Sphagnum* species were grouped into their subgenera: ACUTIFOLIA (*S. fuscum, S. warnstorfii* & *S. capillifolium*), SPHAGNUM (*S. magellanicum*, *S. divinium/medium*, *S. papillosum* & *S. cristatum*), and CUSPIDATA (*S. cuspidatum* & *S. majus*; Table S1). Geographical locations were assigned according to Table S3. Effects of these different variables on the D6^*S*^/D6^*R*^ ratio were assessed by ANOVA, which was done in R (version 3.6.1, RStudio, Inc.) by computing linear mixed effect models using the *lmer*() function of the *lme4* package^67^. To perform ANOVA on the random effect and the fixed effects, the functions *ranova*() and *anova*() were applied respectively. Post-hoc Fisher’s LSD tests with Benjamini–Hochberg correction were computed (in Fig. S1), using the *LSD.test*() function, to account for false discovery rates^68^. To test for multicollinearity among the variables, we calculated the variance inflation factors (VIF) using the *vif*() function of the *car* package. All variables/factors and interaction terms showed a VIF of less than five (range 2.7–4.3), indicating no significant collinearity^69^. Effect of changes in WT, temperature and precipitation were tested by ANOVA, by computing linear models using the *lm*() function and automated stepwise model selection based on Akaike’s information criterion using the *step*() function of the *stats* package^70^. PCA was performed by using the SIMCA-P software package version 16 (Umetrics, Umeå, Sweden). Biplots were created by using the biplot function, which displays both PCA scores, and the correlation scaled loadings of principle component 1 and 2. All other statistical analyses were performed in Excel. Student’s t-tests were one-tailed assuming unequal variance.

### Climate data analysis

Climate data such as annual temperature and precipitation as well as summer temperature and precipitation (June–August and December–February for northern and southern hemisphere, respectively) were obtained from existing climate reconstruction data^33–35^. Changes during the twentieth century were estimated by calculating the 3-year averages around the approximate ages (± 1 year) estimated for the modern and historical samples (Table S5). Luterbacher et al.^33^ and Pauling et al.^34^ provide a dataset of seasonal (3-month) mean air temperatures and total precipitation levels respectively, for Europe (defined as the region between 35.25°N and 69.75°N, and 24.75°W and 39.75°E), covering the period from 1500 to 2002 with a 0.5° gridded resolution (https://crudata.uea.ac.uk/cru/projects/soap/data/). Willmott and Matsuura^35^ provide a global dataset of mean monthly air temperatures and total precipitation from 1900 to 2017 with a 0.5° gridded resolution (http://climate.geog.udel.edu/~climate). Consequently, the Willmott and Matsuura dataset^35^ was used to estimate the climate after 1900 and the Luterbacher et al. and Pauling et al. dataset^33,34^ to estimate the climate before 1900. The two databases showed differences specifically for the Italian sites; therefore, data for 1860 and 1880 were normalized against the more recent data (1990–2000) of Willmot and Matsuura^35^. Australian precipitation data from before 1900 were obtained from the Bureau of Meteorology, Australian Government, 2020 (http://www.bom.gov.au/climate/data). Temperature data were not available for the Australian site and were therefore estimated for 1900 using the Willmott and Matsuura dataset^35^. The southern Argentinian samples were estimated to be ≈ 860 years old, thus the temperature and precipitation data for the Argentinian site were estimated according to other climate models^71,72^.

## Supplementary Information

Supplementary Figures.

Supplementary Tables.
