# Supplementary material for: Global CO2 fertilization of Sphagnum peat mosses via suppression of photorespiration during the twentieth century
Source: Sci Rep. 2021 Dec 31;11:24517. doi: 10.1038/s41598-021-02953-1 (PMC8720097; doi:10.1038/s41598-021-02953-1)
Supplement: Supplementary file 1 — Supplementary Figures. [file 41598_2021_2953_MOESM1_ESM.pdf]

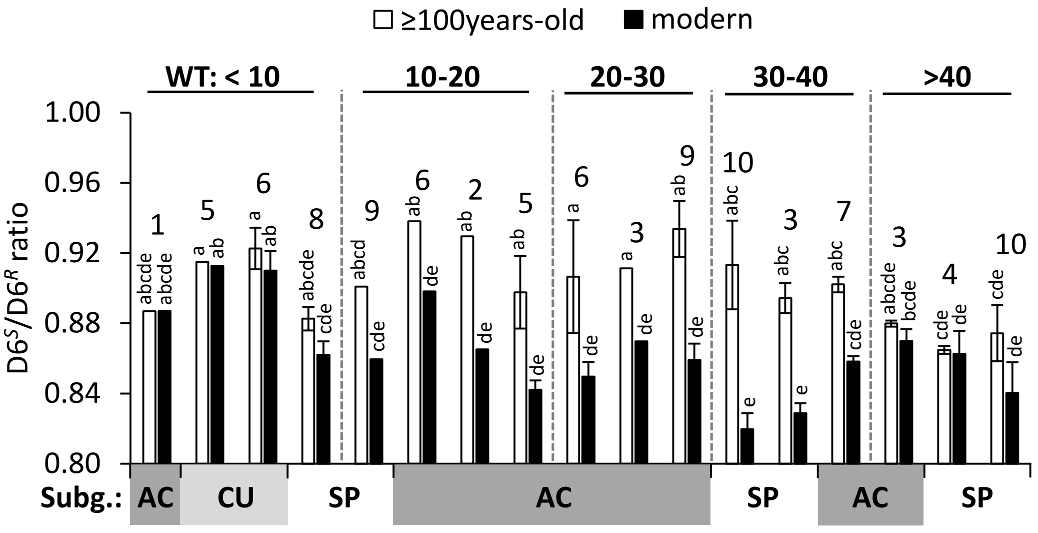

Figure S1. Deuterium isotopomer ($D6^S/D6^R$) ratios of modern and ≥100 years-old *Sphagnum* samples. Five water table depth categories (in cm) are separated by dashed lines. *Sphagnum* subgenera are indicated on the x-axis by grey/white shading: AC, ACUTIFOLIA (dark grey); CU, CUSPIDATA (light grey); SP, SPHAGNUM (white). Error bars indicate standard error, n = 1-4 (see Table S1 for more information). Different letters above error bars indicate significant differences ($p < 0.05$) according to Fisher's least significant difference post-hoc test with Benjamini-Hochberg correction. Numbers above these letters indicate sampling sites according to Figure 1A.

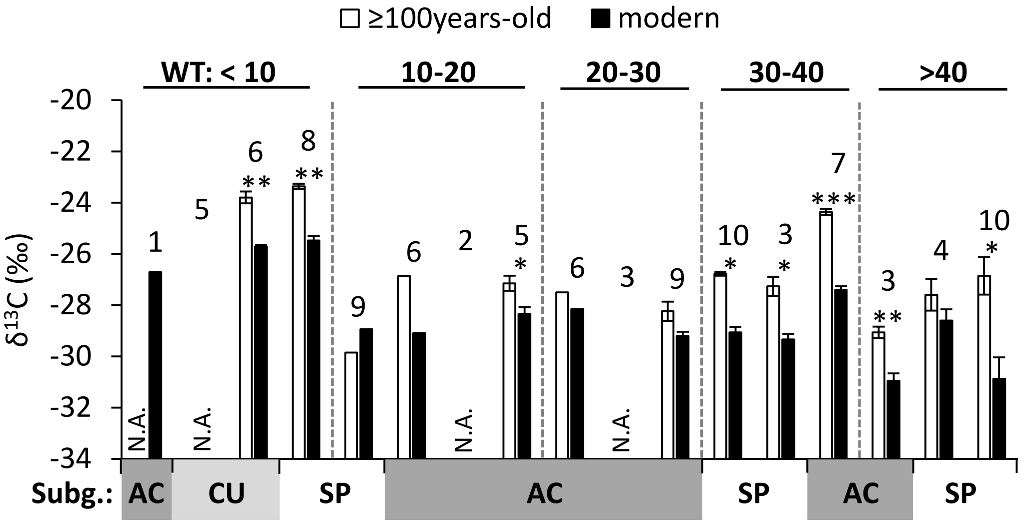

Figure S2. Whole-tissue $\delta^{13}C$ values of modern and ≥100 years-old *Sphagnum* samples. Five water table depth categories (in cm) are separated by dashed lines. *Sphagnum* subgenera are indicated on the x-axis by grey/white shading: AC, ACUTIFOLIA (dark grey); CU, CUSPIDATA (light grey); SP, SPHAGNUM (white). N.A., not analyzed due to insufficient sample material. Error bars indicate standard error, n = 1-4 (see Table S1 for more information). Asterisks above error bars indicate significant difference according to student's t-test: *, $p < 0.05$; **, $p < 0.01$; ***, $p < 0.001$. Numbers above the asterisks indicate sampling sites according to Figure 1A.

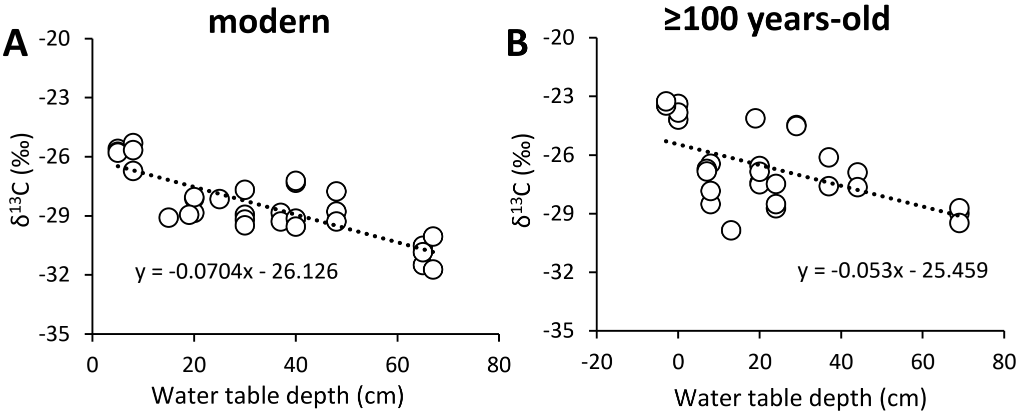

Figure S3. Correlation between recent *Sphagnum* whole-tissue $\delta^{13}C$ values and the present water table depth (modern, A) and between ≥100 years-old *Sphagnum* $\delta^{13}C$ values and the historical water table depth inferred from testate amoebae data reported in the literature (≥100 years-old, B). Lines represent linear regressions with R^2=0.67, p<0.001 and R^2=0.31, p=0.002 for modern and ≥ 100 years-old respectively.

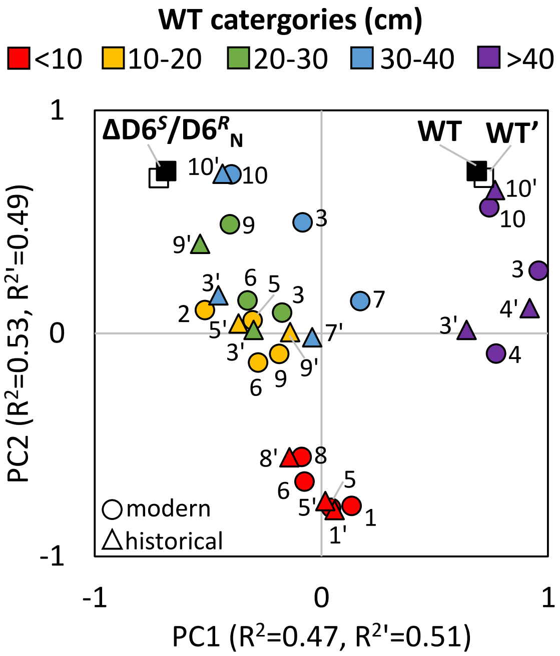

Figure S4. PCA biplots of the deuterium isotopomer ratio ($\Delta D6^{S}/D6^{R}{}_{N}$) of *Sphagnum* during the 20th century and the measured water table depth (WT, circles) and the mean seasonal water table depth reported in the literature (WT', triangles). Colour coding indicates WT categories. Numbers indicate respective sites in Figure 1A. Apostrophe indicates literature data. Note that literature data for site 2 and 6 are missing.
