# Supplementary material for: Global CO2 fertilization of Sphagnum peat mosses via suppression of photorespiration during the twentieth century
Source: Sci Rep. 2021 Dec 31;11:24517. doi: 10.1038/s41598-021-02953-1 (PMC8720097; doi:10.1038/s41598-021-02953-1)
Supplement: Supplementary file 2 — Supplementary Tables. [file 41598_2021_2953_MOESM2_ESM.docx]

**SUPPLEMENTAL TABLES**

**Table S1**. Characteristics of sampled peat cores at different sites, approximate age of *Sphagnum* at the indicated depth, and the corresponding atmospheric CO_2_ concentration.

| **Location** | **Cores** | **Species** | **WT**  **(cm)** | **Micro-**  **form** | **Depth**  **(cm)** | **Year (A.D.)** | **CO_2_**  **(ppm)** |
| --- | --- | --- | --- | --- | --- | --- | --- |
| Northern Canada (1) | 1 | Fus | 8 | Hum | 30 | 1900* | 296 |
| Southern Canada (2) | 1 | War | 15 | Hum | 30 | 1900* | 296 |
| Southern Canada (3) | 3 | Cap | 65 | Hum | 40 | 1900^[73]^ | 296 |
|  | 1 | Fus | 25 | Hum | 30 | 1900* | 296 |
|  | 2 | Div/Med | 40 | Lawn | 40 | 1900^[73]^ | 296 |
| Southern Argentina (4) | 3 | Mag | 48 | Hum | 60 | 1150^[74]^ | 280 |
| Northern Sweden (5) | 3  +1 | Fus  Fus | 20  N.A. | Hum  Hum | 30  N.A. | 1880^[37]^  1921^[26]^ | 291  304 |
|  | 1 | Maj | 5 | Lawn | 30 | 1934* | 309 |
| Southern Sweden (6) | 1  +2 | Fus  Fus | 25  N.A. | Hum  Hum | 40  N.A. | 1880^[37]^ 1888^[26]^  1921^[26]^ | 291  294  305 |
|  | 1 | Cap | 15 | Hum | 30 | 1920^[37]^ | 303 |
|  | 3 | Cus | 5 | Lawn | 2 x 30  1 x 40 | 1920^[37]^  1880^[37]^ | 303  291 |
| Northern Italy (7) | 3 | Fus | 37 | Hum | 40 | 1860^[26]^ | 286 |
| Northern Italy (8) | 2 | Pap | 8 | Lawn | 40 | 1880^[26]^ | 291 |
| Northern China (9) | 3 | Fus | 30 | Hum | 32 | 1915^[43]^ | 302 |
|  | 1 | Div/Med | 19 | Lawn | 32 | 1915^[43]^ | 302 |
| Southern Australia (10) | 2 | Cri | 67 | Hum | 38 | 1890^[61]^ | 294 |
|  | 2 | Cri | 37 | Lawn | 38 | 1890^[61]^ | 294 |

Species: *Sphagnum*: Fus, *fuscum*; War, *warnstorfii*; Cap, *capillifolium*; Cri, *cristatum*; Mag, *magellanicum*; Div/Med, *divinium/medium*; Maj, *majus*; Cus, *cuspidatum*; Pap, *papillosum*. Cores, number of replicate peat cores. WT, water table depth at time of sampling in cm below moss surface. Hum, Hummock. Depth, sampling depth of historical tissues in cm. * indicates age determined by ^210^Pb dating. All other ages were estimated according to age-depth profiles from literature. +1 and +2 indicate the number of herbarium samples from Ehlers et al. (2015) ^[26]^ included in this study.

**Table S2.** Coordinates, distance and elevation of mires from the same region as the indicated site, used for estimation of historical changes in water table in Table 2.

| **Mire name (site)** | **Coordinates** | **Distance**  **to site (km)** | **Elevation**  **(m asl)** | **Reference** |
| --- | --- | --- | --- | --- |
| Andorra bog (4) | 54⁰45’S 68⁰20’W | 11 | 200 | [38] |
| Lappmyran (5) | 64⁰10’N 19⁰35’E | 53 | 285 | [37] |
| Undarsmosse (6) | 56⁰48’N 12⁰39’E | 170 | 20 | [42] |
| Mauntschas bog (7) | 46⁰29’N 9⁰51’E | 95 | 1825 | [37] |
| Snowy Flat bog (10) | 35⁰34’S 148⁰47’E | 277 | 1610 | [44] |

**Table S3**. Coordinates and climate data of sites from which studied peat cores were taken.

| Site | Co-ordinates | Climate | Mire  type | | MAT (°C) | MAP (mm) | Elev.  (m asl) | Reference |
| --- | --- | --- | --- | --- | --- | --- | --- | --- |
| Northern Canada (1) | 61°18'N 121°18'W | Dfc | | thermo-karst bog | -3.2 | 369 | 285 | [75] |
| Southern Canada  (2) | 45°35'N 76°00′W | Dfb | | peat swamp forest | 5.9 | 980 | 133 | [76] |
| Southern Canada  (3) | 45°24′N 75°30′W | Dfb | | ombro-trophic bog | 6.0 | 943 | 69 | [49] |
| Southern Argentina (4) | 54˚49'S 68˚27'W | ET | | ombro-trophic bog | 5.0 | 487 | 90 | [77] |
| Northern Sweden (5) | 63°44’N 20°06’E | Dfc | | string bog (aapa) | 4.0 | 510 | 35 | [78] |
| Southern Sweden (6) | 56°56’N 15°25’E | Dfb | | poor fen | 7 | 800 | 255 | [26] |
| Northern Italy (7) | 46°28'N 11°04'E | Dfc | | ombro-trophic bog | 4.9 | 825 | 1700 | [79] |
| Northern Italy (8) | 46°26'N 11°24'E | Dfb | | ombro-trophic bog | 6.3 | 810 | 1300 | [79] |
| Northern China (9) | 52°56'N 122°51'E | Dwc | | poor fen | -3.9 | 452 | 473 | [80] |
| Southern Australia (10) | 37˚30'S 146˚50'E | Cfb | | ombro-trophic bog | 5-6 | 1270 | 1480 | [61] |

Climate, Köppen climate classification according to Kottek et al. (2006) ^[81]^. MAT, mean annual air temperature; MAP, mean annual precipitation; Elev., elevation.

**Table S4**. Compilation of published data on growing season average water table depth for the respective sites.

| **Site** | **Water table (cm)** | **Comment** | **Reference** |
| --- | --- | --- | --- |
| Northern Canada (1) | 8 | Testate amoebae | [38] |
| Southern Canada (3) | 20  45 (hummock) | Testate amoebae;  Mean WT  (June-Sept, 1998-2009) | [39], [40]  [82] |
| Southern Argentina (4) | 60  50; <40 | Testate amoebae;  direct measurements | [41]  [77], [62] |
| Northern Sweden (5) | 19 (hummock)  8 (lawn) | Mean WT  (May-Sept 1992-1993) | [83] |
| Northern  Italy (7) | 26 | Mean WT  (May-Sept 2015-2016) | [79] |
| Northern  Italy (8) | 9 | Mean WT  (summer 1999) | [84] |
| Northern  China (9) | 20  28 | Testate amoebae;  Mean WT  (May-Oct, 2010-2011) | [43]  [85] |
| Southern Australia (10) | 65 | direct measurement | [61] |

**Table S5**. Estimated year, CO_2_ concentration, mean annual air temperature (MST), mean summer temperature (MST), total annual precipitation (TAP), and total summer precipitation (TSP) for modern and ≥100 years-old *Sphagnum* samples from peat cores listed in Table S1.

|  | **Modern** | | | | | | **≥100 years-old** | | | | | | | | | | | |
| --- | --- | --- | --- | --- | --- | --- | --- | --- | --- | --- | --- | --- | --- | --- | --- | --- | --- | --- |
| **Location** | Year  (AD) | CO_2_  (ppm) | MAT  (°C) | MST  (°C) | TAP  (mm) | TSP  (mm) | Year  (AD) | CO_2_  (ppm) | | | MAT  (°C) | | MST  (°C) | TAP  (mm) | | TSP  (mm) | |  |
| Northern Canada (1) | 2014 | 399 | -2.5 | 16.7 | 367.3 | 165.6 | 1900 | | 296 | -5.6 | | 13.2 | | | 330.9 | | 96 | |
| Southern Canada (2) | 2014 | 399 | 6.5 | 19.8 | 793.3 | 262.5 | 1900 | | 296 | 6.1 | | 19.5 | | | 889.4 | | 275 | |
| Southern Canada (3) | 2016 | 405 | 6.7 | 19.9 | 934.7 | 293.0 | 1900 | | 296 | 6.4 | | 19.5 | | | 996.4 | | 299 | |
|  | 2004 | 378 | 6.2 | 19.3 | 945.8 | 268.1 | 1900 | | 296 | 6.4 | | 19.5 | | | 996.4 | | 299 | |
|  | 2016 | 405 | 6.7 | 19.9 | 934.7 | 293.0 | 1900 | | 296 | 6.4 | | 19.5 | | | 996.4 | | 299 | |
| Southern Argentina (4) | 2014 | 399 | 4.3 | 7.3 | 404.6 | 124.1 | 1150 | | 280 | 6.0 | | 9.5 | | | 600 | | 156 | |
| Northern Sweden (5) | 2015 | 401 | 4.6 | 14.8 | 650.3 | 160.5 | 1890 | | 294 | 1.2 | | 12.5 | | | 484.9 | | 169 | |
|  | 2006 | 382 | 4.6 | 14.9 | 652.3 | 196.3 | 1934 | | 309 | 4.1 | | 14.5 | | | 697.3 | | 163.2 | |
| Southern Sweden (6) | 2013 | 397 | 7.0 | 16.2 | 652.5 | 250.6 | 1897 | | 297 | 5.0 | | 14.1 | | | 655.6 | | 213.1 | |
|  | 2013 | 397 | 6.8 | 16.4 | 682 | 284.2 | 1920 | | 303 | 6.5 | | 15 | | | 539.7 | | 171.1 | |
|  | 2013 | 397 | 6.8 | 16.4 | 682 | 284.2 | 1907 | | 299 | 5.9 | | 14.9 | | | 566.8 | | 177.6 | |
| Northern Italy (7,8) | 2016 | 405 | 4.4 | 12.4 | 1165.3 | 333.4 | 1860 | | 286 | 4.4 | | 11.1 | | | 952.4 | | 264.4 | |
|  | 2016 | 405 | 7.7 | 16.3 | 889.6 | 291.2 | 1880 | | 291 | 6.7 | | 14.5 | | | 855.6 | | 265.5 | |
| Northern China (9) | 2017 | 407 | -4.6 | 15.3 | 576.3 | 301.3 | 1915 | | 302 | -6.2 | | 14.8 | | | 432 | | 306.7 | |
|  | 2017 | 407 | -4.6 | 15.3 | 576.3 | 301.3 | 1915 | | 302 | -6.2 | | 14.8 | | | 432 | | 306.7 | |
| Southern Australia (10) | 2018 | 409 | 7.4 | 13.7 | 983.7 | 124.1 | 1890 | | 294 | 7.2 | | 13.7 | | | 835.2 | | 208.1 | |
|  | 2018 | 409 | 7.4 | 13.7 | 983.7 | 124.1 | 1890 | | 294 | 7.2 | | 13.7 | | | 835.2 | | 208.1 | |
|  |  |  |  |  |  |  |  | |  |  | |  | | |  | |  | |

**ADDITIONAL REFERENCES**

73. Bilali H.E., Patterson R.T., Prokoph A. (2013). A Holocene paleoclimate reconstruction for eastern Canada based on δ^18^O cellulose of *Sphagnum* mosses from Mer Bleue Bog. *The Holocene* 23(9): 1260-1271.

74. Borromei A.M., Musotto L.L., Coronato A., Ponce J.F., Pontevedra-Pombal X. (2016). Postgracial vegetation and climate changes inferred from a peat pollen record in the Río Pipo valley, southern Tierra del Fuego. *APA* 16(2): 168-183.

75. Quinton W.L., Baltzer J.L. (2013). The active-layer hydrology of a peat plateau with thawing permafrost (Scotty Creek, Canada). *Hydrogeology Journal* 21: 201-220.

76. Blarquez O., Talbot J., Paillard J., Lapointe-Elmrabti L., Pelletier N., St-Pierre C.G. (2018). Late Holocene influence of societies on the fire regime in southern Québec temperate forests. *Quaternary Science Reviews* 180: 63-74.

77. Lehmann J.R.K., Münchberger W., Knoth C., Blodau C., Nieberding F., Prinz T., Pancotta V.A., & Kleinbecker T. (2016). High-resolution classification of south Patagonian peat bog microforms reveals potential gaps in up-scaled CH_4_ fluxes by use of unmanned aerial system (UAS) and CIR imagery. *Remote Sensing* 8(173), doi:10.3390/rs8030173.

78. Bohdalkova L., Novak M., Buzek F., Kreisinger J., Bindler R., Pazderu K., Pacherova P. (2014). The response of a mid- and high latitude peat bog to predicted climate change: methane production in a 12-month peat incubation. *Mitigation and Adaptation Strategies for Global Change* 19: 997-1010.

79. Zeh L., Limpens J., Erhagen B., Bragazza L., Kalbitz K. (2019). Plant functional types and temperature control carbon input via roots in peatland soils. *Plant Soil* 438: 19-38.

80. Yu X., Song C., Sun L., Wang X., Shi F., Cui Q., Tan W. (2017). Growing season methane emissions from a permafrost peatland of northeast China: Observations using open-path eddy covariance method. *Atmospheric Environment* 153: 135-149.

81. Kottek, M., Grieser J., Beck C., Rudolf B., & Rubel F. (2006). World map of the Köppen-Geiger climate classification. *Meteorologische Zeitschrift* 15, 259-263. [doi: 10.1127/0941-2948/2006/0130](https://web.archive.org/web/20100906034159/http:/dx.doi.org/10.1127/0941-2948/2006/0130).

82. Wilson, P.G. (2012). The relationship among micro-topographic variation, water table depth and biogeochemistry in an ombrotrophic bog. Master thesis. Department of Geography, McGill University, Montreal, Canada.

83. Waddington, J.M. & Roulet, T. (2000). Carbon balance of a boreal patterned peatland. *Global Change Biology* 6: 87-97.

84. Bragazza, L., Rydin, H., & Gerdol, R. (2005). Multiple gradients in mire vegetation: a comparison of a Swedish and an Italian bog. *Plant Ecology* 177: 223-236.

85. Miao, Y., Song, C., Sun, L., Wang, X., Meng, H., & Mao, R. (2012). Growing season methane emission from a boreal peatland in the continuous permafrost zone of Northeast China: effects of active layer depth and vegetation. *Biogeosciences* 9: 4455-4464. doi:10.5194/bg-9-4455-2012.
